# Methyl *c*-1-cyano-*t*-2-methyl­sulfonyl-3-phenyl­cyclo­propane­carboxyl­ate

**DOI:** 10.1107/S1600536811016370

**Published:** 2011-05-25

**Authors:** Victor A. Vasin, Irina Yu. Bolusheva, Vyacheslav A. Neverov, Nikolai V. Somov

**Affiliations:** a Department of Chemistry, N. P. Ogarev Mordovian State University, 430005 Saransk, Russian Federation; b Department of Physics, N. P. Ogarev Mordovian State University, 430005 Saransk, Russian Federation; c Department of Physics, N. I. Lobachevsky State University of Nizhni Novgorod, 603950 Nizhni Novgorod, Russian Federation

## Abstract

The title compound, C_13_H_13_NO_4_S, is a racemic mixture of enanti­omers. Short intra­molecular contacts between sulfonyl O and ester carbonyl C atoms are observed [C⋯O = 2.881 (1), 2.882 (1) and 2.686 (1) Å], indicating the possibility of donor—acceptor inter­actions between these groups. The dihedral angle between the phenyl and cyclopropyl rings is 79.3 (1)°.

## Related literature

Some α-bromo­vinyl sulfones react with primary amines in DMSO to give the products of aza-Michael ring closure reactions (MIRCR), *viz*. 2-sulfonyl-substituted aziridines, see: Galliot *et al.* (1979 ▶). Similarly, MIRCR of phenyl-(*Z*)-(2-phenyl-2-chloro­ethen­yl)sulfone with diethyl sodium malonate leads to the formation of a sulfonyl-substituted cyclo­propane, see: Yamamoto *et al.* (1985 ▶). For related structures, see: Vasin *et al.* (2008 ▶, 2010 ▶); Zefirov & Zorkii (1989 ▶).
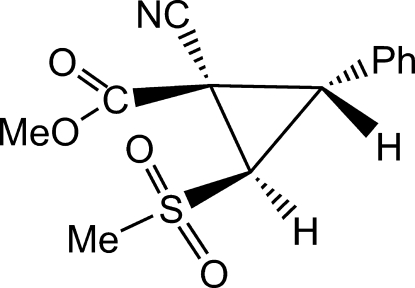


## Experimental

### 

#### Crystal data

C_13_H_13_NO_4_S
*M* *_r_* = 279.3
Orthorhombic, 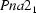

*a* = 10.7323 (4) Å
*b* = 20.0790 (6) Å
*c* = 6.2663 (2) Å
*V* = 1350.35 (8) Å^3^
*Z* = 4
Mo *K*α radiation
μ = 0.25 mm^−1^
*T* = 293 K
0.20 × 0.15 × 0.12 mm

#### Data collection

Xcalibur, Sapphire3, Gemini diffractometer
Absorption correction: multi-scan (*CrysAlis PRO*; Oxford Diffraction, 2010 ▶) *T*_min_ = 0.964, *T*_max_ = 1
21704 measured reflections
3353 independent reflections
3044 reflections with *I* > 2σ(*I*)
*R*_int_ = 0.026

#### Refinement

*R*[*F* ^2^ > 2σ(*F* ^2^)] = 0.031
*wR*(*F* ^2^) = 0.078
*S* = 0.98
3353 reflections
172 parameters
1 restraint
H-atom parameters constrained
Δρ_max_ = 0.23 e Å^−3^
Δρ_min_ = −0.14 e Å^−3^
Absolute structure: Flack (1983 ▶), 1523 Friedel pairs
Flack parameter: 0.05 (5)

### 

Data collection: *CrysAlis PRO* (Oxford Diffraction, 2010 ▶); cell refinement: *CrysAlis PRO*; data reduction: *CrysAlis PRO*; program(s) used to solve structure: *SHELXS97* (Sheldrick, 2008 ▶); program(s) used to refine structure: *SHELXL97* (Sheldrick, 2008 ▶); molecular graphics: *PLATON* (Spek, 2009 ▶); software used to prepare material for publication: *publCIF* (Westrip 2010 ▶).

## Supplementary Material

Crystal structure: contains datablocks global, I. DOI: 10.1107/S1600536811016370/aa2006sup1.cif

Structure factors: contains datablocks I. DOI: 10.1107/S1600536811016370/aa2006Isup2.hkl

Supplementary material file. DOI: 10.1107/S1600536811016370/aa2006Isup3.cml

Additional supplementary materials:  crystallographic information; 3D view; checkCIF report

## Figures and Tables

**Fig. 1.**
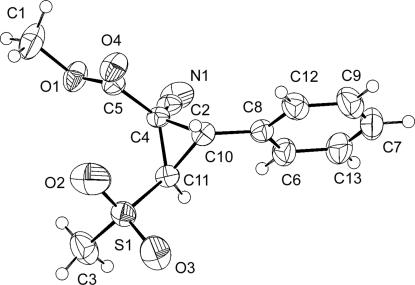
A view of the compound (2). The non-H atoms are shown with displacement ellipsoids drawn at the 50% probability level.

**Fig. 2.**
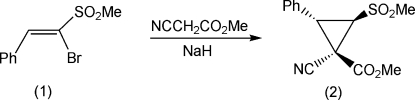
MIRCR between compound (1) and monosodium salt of methyl cyanoacetate in THF

## supplementary crystallographic information

### Comment

It is know, that some alpha-bromovinyl sulfones react with primary amines in DMSO to give the products of aza-Michael Ring Closure reaction (MIRCR) - 2-sulfonylsubstituted aziridines (Galliot *et al.*, 1979). Similarly MIRCR of phenyl-(*Z*)-(2-phenyl-2-chloroethenyl)sulfone with diethyl sodium malonate leads to formation of a sulfonylsubstituted cyclopropane (Yamamoto *et al.*, 1985). We have carried out MIRCR between compound (1) (see Fig. 2) and monosodium salt of methyl cyanoacetate in THF at 20 °C and a cyclopropane derivative, (2), was obtained. The product, (2), was isolated by chromatography, crystallized and studied by X-ray diffraction.

In compound (2) three short intramolecular contacts C···O, which appreciable less sum of the van der Vaals radii given atoms = 3.000Å (Zefirov *et al.*, 1989), are found out. The first of them takes place between atoms O2 of sulfonyl and C5 of methoxycarbonyl groups (2.881 Å). The second contact length 2.882Å is observed between atoms O4 of methoxycarbonyl and C10 of cyclopropane fragment. The third is shorted (2.686 Å). It arises between atoms O1 of methoxycarbonyl group and C2 of cyano group. Given contacts are evidence of possible donor-acceptor interaction between *cys*-located sulfonyl and methoxycarbonyl groups, and also between methoxycarbonyl on the one hand,cyano group and cyclopropene fragment - with another.

We shall note, that strong interaction between drawing together sulfonyl and methoxycarbonyl groups in structure of cyclobutane fragment, hardly fixed in space by trimethylene bridge, where free rotation of given groups was revealed earlier; interatomic distance C···O in this case is 2.489Å (Vasin *et al.*, 2010). At the same time, in analogue of compound (2) - dimethyl 3-phenyl-2-(*t*)-phenylsulfonyl-1,1-cyclopropanecarboxylate, as it has been established by X-ray analysis, mutual, close to parallel, an arrangement of sulfonyl and ethoxycarbonyl groups the dipole-dipole interaction between them does not promote (Yamamoto *et al.*, 1985).

The values of valent angles at atom C5 in compound (2), most likely, are consequence of noted donor-acceptor interaction: a little overestimated for C4—C5—O4 (124.35°) and O4—C5—O1 (126.02°), essentially underestimated for O1—C5—C4 (109.62°), and also value of angle C5—C4—C2 (114.35°).

The structure of compound (2) consists of separate molecules between which only van der Vaals interaction is carried out.

### Experimental

Compound (2) was obtained by the reaction between the previously reported compound (1) (Vasin *et al.*, 2008) and monosodium salt of methyl cyanoacetate (see Fig. 2). Sodium hydride (0.23 g 60% suspension in mineral oil) was freed of mineral oil by washing with hexane and was added dry THF (5 ml). Methyl cyanoacetate (4.2 g) in THF (10 ml) was added drop wise for 10 min at stirring. The stirring was continued for 1.5 h at 20 °C. After drop wise addition of compound (1) (1.0 g) in THF (10 ml), stirring was continued at 20° C for 20 h. The mixture was diluted with water (250 ml), neutralized with aqueous (1: 1) HCl, extracted with CHCl_3_ (3 *x* 15 ml), washed with water, and dried over MgSO_4_. Evaporation of solvent *in vacuo* gave 0.7 g semisolid product. Compound (2) was isolated by column chromatography on silicagel and crystallized from an acetone - hexane (1: 3) mixture [yield 0.21 g (20%); m. p. 417–418 K].

### Refinement

The initial fragment of structure was solved by a direct method; other non-hydrogen atoms were received from the analysis by successive synthesis of electron density. Floating origin restraint had been used. Hydrogen atoms were placed in geometrically calculated positions and refined in riding model with U (H) = 1.5 U (C) for hydrogen atoms in methyl groups and U (H) = 1.2 U (C) for all other hydrogen atoms, where U (C) – the equivalent temperature factor of carbon atom with which the corresponding hydrogen atom is bonded.

### Crystal data

**Table:**

C_13_H_13_NO_4_S	*F*(000) = 584
*M**_r_* = 279.3	*D*_x_ = 1.374 Mg m^−^^3^
Orthorhombic, *P**n**a*2_1_	Mo *K*α radiation, λ = 0.71073 Å
Hall symbol: P 2c -2n	Cell parameters from 10874 reflections
*a* = 10.7323 (4) Å	θ = 3.4–32.9°
*b* = 20.0790 (6) Å	µ = 0.25 mm^−^^1^
*c* = 6.2663 (2) Å	*T* = 293 K
*V* = 1350.35 (8) Å^3^	Prism, colorless
*Z* = 4	0.20 × 0.15 × 0.12 mm

### Data collection

**Table:**

Xcalibur, Sapphire3, Gemini diffractometer	3353 independent reflections
graphite	3044 reflections with *I* > 2σ(*I*)
Detector resolution: 16.0302 pixels mm^-1^	*R*_int_ = 0.026
ω scans	θ_max_ = 28.3°, θ_min_ = 3.4°
Absorption correction: multi-scan (*CrysAlis PRO*; Oxford Diffraction, 2010)	*h* = −14→14
*T*_min_ = 0.964, *T*_max_ = 1	*k* = −26→26
21704 measured reflections	*l* = −8→8

### Refinement

**Table:**

Refinement on *F*^2^	Secondary atom site location: difference Fourier map
Least-squares matrix: full	Hydrogen site location: inferred from neighbouring sites
*R*[*F*^2^ > 2σ(*F*^2^)] = 0.031	H-atom parameters constrained
*wR*(*F*^2^) = 0.078	*w* = 1/[σ^2^(*F*_o_^2^) + (0.0559*P*)^2^] where *P* = (*F*_o_^2^ + 2*F*_c_^2^)/3
*S* = 0.98	(Δ/σ)_max_ < 0.001
3353 reflections	Δρ_max_ = 0.23 e Å^−^^3^
172 parameters	Δρ_min_ = −0.14 e Å^−^^3^
1 restraint	Absolute structure: Flack (1983), 1523 Friedel pairs
Primary atom site location: structure-invariant direct methods	Flack parameter: 0.05 (5)

### Special details

**Table:**

Experimental. CrysAlisPro (Oxford Diffraction Ltd., 2010) Empirical absorption correction using spherical harmonics, implemented in SCALE3 ABSPACK scaling algorithm.
Geometry. All s.u.'s (except the s.u. in the dihedral angle between two l.s. planes) are estimated using the full covariance matrix. The cell s.u.'s are taken into account individually in the estimation of s.u.'s in distances, angles and torsion angles; correlations between s.u.'s in cell parameters are only used when they are defined by crystal symmetry. An approximate (isotropic) treatment of cell s.u.'s is used for estimating s.u.'s involving l.s. planes.
Refinement. The one restraint corresponded to floating origin restraints. Refinement of *F*^2^ against ALL reflections. The weighted *R*-factor *wR* and goodness of fit *S* are based on *F*^2^, conventional *R*-factors *R* are based on *F*, with *F* set to zero for negative *F*^2^. The threshold expression of *F*^2^ > σ(*F*^2^) is used only for calculating *R*-factors(gt) *etc*. and is not relevant to the choice of reflections for refinement. *R*-factors based on *F*^2^ are statistically about twice as large as those based on *F*, and *R*- factors based on ALL data will be even larger.

### Fractional atomic coordinates and isotropic or equivalent isotropic displacement parameters (Å^2^)

**Table:**

	*x*	*y*	*z*	*U*_iso_*/*U*_eq_	
S1	0.29436 (2)	0.295171 (11)	0.76310 (4)	0.04011 (6)	
O1	−0.01457 (7)	0.35848 (4)	1.05436 (11)	0.04777 (19)	
O2	0.27636 (12)	0.27721 (5)	0.98035 (16)	0.0874 (4)	
O3	0.41644 (8)	0.29350 (4)	0.67581 (19)	0.0666 (3)	
O4	0.16184 (7)	0.38091 (4)	1.22923 (10)	0.04745 (19)	
N1	−0.03319 (10)	0.47196 (5)	0.65545 (16)	0.0532 (3)	
C1	−0.07127 (13)	0.33421 (7)	1.2490 (2)	0.0652 (3)	
H1A	−0.1541	0.3188	1.2191	0.098*	
H1B	−0.0749	0.3696	1.3519	0.098*	
H1C	−0.0225	0.2982	1.3052	0.098*	
C2	0.04443 (9)	0.44609 (4)	0.74669 (15)	0.0345 (2)	
C3	0.20022 (12)	0.24604 (6)	0.5992 (3)	0.0643 (4)	
H3A	0.1166	0.2460	0.6533	0.097*	
H3B	0.2317	0.2013	0.5971	0.097*	
H3C	0.2007	0.2638	0.4569	0.097*	
C4	0.14145 (8)	0.41098 (4)	0.86151 (14)	0.02960 (19)	
C5	0.10008 (9)	0.38160 (5)	1.07244 (13)	0.0337 (2)	
C6	0.28383 (11)	0.51912 (5)	0.55263 (17)	0.0454 (3)	
H6	0.2305	0.4930	0.4707	0.054*	
C7	0.40648 (13)	0.61805 (6)	0.5922 (2)	0.0642 (4)	
H7	0.4357	0.6582	0.5376	0.077*	
C8	0.31902 (8)	0.49822 (4)	0.75425 (17)	0.0354 (2)	
C9	0.44182 (12)	0.59803 (6)	0.7918 (2)	0.0617 (4)	
H9	0.4947	0.6247	0.8726	0.074*	
C10	0.27718 (8)	0.43416 (5)	0.85051 (15)	0.0327 (2)	
H10	0.3262	0.4211	0.9757	0.039*	
C11	0.23653 (9)	0.37605 (5)	0.71750 (14)	0.0319 (2)	
H11	0.2267	0.3868	0.5659	0.038*	
C12	0.39901 (11)	0.53807 (5)	0.8741 (2)	0.0475 (3)	
H12	0.4238	0.5245	1.0095	0.057*	
C13	0.32830 (14)	0.57925 (6)	0.4726 (2)	0.0600 (3)	
H13	0.3047	0.5932	0.3369	0.072*	

### Atomic displacement parameters (Å^2^)

**Table:**

	*U*^11^	*U*^22^	*U*^33^	*U*^12^	*U*^13^	*U*^23^
S1	0.03721 (11)	0.03399 (9)	0.04912 (13)	0.00838 (9)	0.00024 (12)	0.00378 (12)
O1	0.0483 (4)	0.0622 (4)	0.0328 (3)	−0.0185 (3)	−0.0033 (3)	0.0071 (3)
O2	0.1419 (10)	0.0668 (5)	0.0534 (5)	0.0470 (6)	0.0050 (6)	0.0223 (5)
O3	0.0311 (4)	0.0541 (4)	0.1144 (8)	0.0099 (3)	0.0021 (4)	0.0004 (5)
O4	0.0514 (4)	0.0653 (4)	0.0257 (3)	−0.0055 (4)	−0.0068 (3)	0.0087 (3)
N1	0.0513 (5)	0.0676 (6)	0.0406 (4)	0.0219 (5)	−0.0035 (4)	0.0105 (4)
C1	0.0665 (7)	0.0860 (8)	0.0431 (6)	−0.0335 (6)	0.0020 (6)	0.0121 (6)
C2	0.0395 (4)	0.0375 (4)	0.0265 (4)	0.0062 (3)	0.0006 (4)	−0.0002 (4)
C3	0.0489 (7)	0.0460 (6)	0.0982 (10)	−0.0072 (5)	−0.0028 (7)	−0.0096 (7)
C4	0.0331 (4)	0.0326 (4)	0.0230 (4)	0.0039 (3)	−0.0016 (4)	0.0010 (3)
C5	0.0418 (5)	0.0348 (4)	0.0245 (4)	0.0006 (4)	−0.0005 (4)	0.0006 (4)
C6	0.0544 (6)	0.0414 (5)	0.0404 (5)	−0.0058 (5)	0.0006 (5)	0.0068 (4)
C7	0.0624 (7)	0.0420 (5)	0.0882 (9)	−0.0070 (6)	0.0216 (7)	0.0101 (6)
C8	0.0339 (4)	0.0359 (4)	0.0365 (4)	0.0011 (3)	0.0024 (4)	−0.0001 (5)
C9	0.0505 (6)	0.0458 (5)	0.0888 (10)	−0.0107 (5)	0.0006 (7)	−0.0115 (6)
C10	0.0318 (4)	0.0376 (5)	0.0286 (4)	0.0012 (4)	−0.0045 (4)	0.0035 (4)
C11	0.0328 (4)	0.0343 (4)	0.0287 (4)	0.0064 (4)	−0.0018 (3)	0.0028 (3)
C12	0.0440 (5)	0.0464 (5)	0.0521 (6)	−0.0034 (5)	−0.0037 (5)	−0.0062 (5)
C13	0.0739 (8)	0.0492 (6)	0.0569 (7)	0.0020 (6)	0.0098 (6)	0.0166 (6)

### Geometric parameters (Å, °)

**Table:**

S1—O3	1.4202 (9)	C4—C11	1.5320 (12)
S1—O2	1.4215 (10)	C6—C8	1.3838 (15)
S1—C3	1.7462 (14)	C6—C13	1.3918 (17)
S1—C11	1.7619 (9)	C6—H6	0.9300
O1—C5	1.3199 (12)	C7—C9	1.367 (2)
O1—C1	1.4477 (14)	C7—C13	1.368 (2)
O4—C5	1.1853 (11)	C7—H7	0.9300
N1—C2	1.1360 (13)	C8—C12	1.3934 (15)
C1—H1A	0.9600	C8—C10	1.4899 (13)
C1—H1B	0.9600	C9—C12	1.3879 (16)
C1—H1C	0.9600	C9—H9	0.9300
C2—C4	1.4488 (12)	C10—C11	1.4988 (13)
C3—H3A	0.9600	C10—H10	0.9800
C3—H3B	0.9600	C11—H11	0.9800
C3—H3C	0.9600	C12—H12	0.9300
C4—C5	1.5140 (12)	C13—H13	0.9300
C4—C10	1.5308 (13)		
			
O3—S1—O2	119.22 (7)	C8—C6—H6	120.1
O3—S1—C3	107.09 (6)	C13—C6—H6	120.1
O2—S1—C3	109.97 (8)	C9—C7—C13	120.25 (12)
O3—S1—C11	106.52 (5)	C9—C7—H7	119.9
O2—S1—C11	109.95 (5)	C13—C7—H7	119.9
C3—S1—C11	102.80 (5)	C6—C8—C12	119.08 (9)
C5—O1—C1	115.97 (8)	C6—C8—C10	123.31 (9)
O1—C1—H1A	109.5	C12—C8—C10	117.60 (10)
O1—C1—H1B	109.5	C7—C9—C12	120.20 (12)
H1A—C1—H1B	109.5	C7—C9—H9	119.9
O1—C1—H1C	109.5	C12—C9—H9	119.9
H1A—C1—H1C	109.5	C8—C10—C11	122.32 (9)
H1B—C1—H1C	109.5	C8—C10—C4	124.58 (8)
N1—C2—C4	178.08 (10)	C11—C10—C4	60.74 (6)
S1—C3—H3A	109.5	C8—C10—H10	113.2
S1—C3—H3B	109.5	C11—C10—H10	113.2
H3A—C3—H3B	109.5	C4—C10—H10	113.2
S1—C3—H3C	109.5	C10—C11—C4	60.66 (6)
H3A—C3—H3C	109.5	C10—C11—S1	121.66 (7)
H3B—C3—H3C	109.5	C4—C11—S1	124.13 (6)
C2—C4—C5	114.35 (8)	C10—C11—H11	113.5
C2—C4—C10	120.90 (8)	C4—C11—H11	113.5
C5—C4—C10	115.90 (8)	S1—C11—H11	113.5
C2—C4—C11	114.14 (8)	C9—C12—C8	120.13 (12)
C5—C4—C11	122.09 (7)	C9—C12—H12	119.9
C10—C4—C11	58.60 (6)	C8—C12—H12	119.9
O4—C5—O1	126.04 (9)	C7—C13—C6	120.45 (13)
O4—C5—C4	124.35 (9)	C7—C13—H13	119.8
O1—C5—C4	109.60 (7)	C6—C13—H13	119.8
C8—C6—C13	119.89 (11)		

## References

[bb1] Flack, H. D. (1983). *Acta Cryst.* A**39**, 876–881.

[bb2] Galliot, J.-M., Gellas-Mialhe, Y. & Vessiere, R. (1979). *Can. J. Chem.* **57**, 1958–1966.

[bb3] Oxford Diffraction (2010). *CrysAlis PRO* Oxford Diffraction Ltd, Yarnton, England.

[bb4] Sheldrick, G. M. (2008). *Acta Cryst.* A**64**, 112–122. 10.1107/S0108767307043930 18156677

[bb9] Spek, A. L. (2009). *Acta Cryst.* D**65**, 148–155. 10.1107/S090744490804362X PMC2631630 19171970

[bb5] Vasin, V. A., Bolusheva, I. Yu. & Razin, V. V. (2008). *Chem. Heterocycl. Compd*, **44**, 419–429.

[bb6] Vasin, V. A., Petrov, P. S., Genaev, A. M., Gindin, V. A. & Razin, V. V. (2010). *J. Struct. Chem.* **51**, 949–955.

[bb10] Westrip, S. P. (2010). *J. Appl. Cryst.* **43**, 920–925.

[bb7] Yamamoto, I., Sakai, T., Ohta, K. & Matsuzaki, K. (1985). *J. Chem. Soc. Perkin Trans. 1*, pp. 2785–2787.

[bb8] Zefirov, Yu. V. & Zorkii, P. M. (1989). *Russ. Chem. Rev.* **58**, 421–440.

